# Supplementary material for: Solvent-Producing Clostridia Revisited
Source: Microorganisms. 2023 Sep 7;11(9):2253. doi: 10.3390/microorganisms11092253 (PMC10538166; doi:10.3390/microorganisms11092253)
Supplement: Supplementary file 1 [file microorganisms-11-02253-s001.zip › microorganisms-2519127-supplementary/Supplamentary-Files/Fig.S1 Legend.pdf]

Supplementary Data.

Figure S1. Phylogenomics based evolutionary history of the genus *Clostridium*. Maximum likelihood phylogenetic tree (LG4X+F) of the genus *Clostridium* after adding the 270 genomes from the DJ culture collection (highlighted with red circles). Support values are indicated if below 100. Scale bar indicates the average number of substitutions per site. Pairwise average nucleotide identities (ANI) of 92% and above are displayed next to the phylogenetic tree. All major branches leading to species-level clades were fully supported (support value = 100). Strain names and within species clade support values are shown in the detailed tree. Species were classified using genome-wide ANI comparisons with Illumina sequences and the phylogenetic tree was constructed using a concatenated alignment of 118 single-copy panorthologs that were least affected by horizontal gene transfer (IQ-Tree, GRT+R10).
