# Supplementary figures and images for: Solvent-Producing Clostridia Revisited
Source: Microorganisms. 2023 Sep 7;11(9):2253. doi: 10.3390/microorganisms11092253 (PMC10538166; doi:10.3390/microorganisms11092253)

bootstrap

- 13
- 34
- 55
- 76
- 97

ANI%

0.94  
0.946  
0.952  
0.958  
0.964  
0.97  
0.976  
0.982  
0.988  
0.994  
1

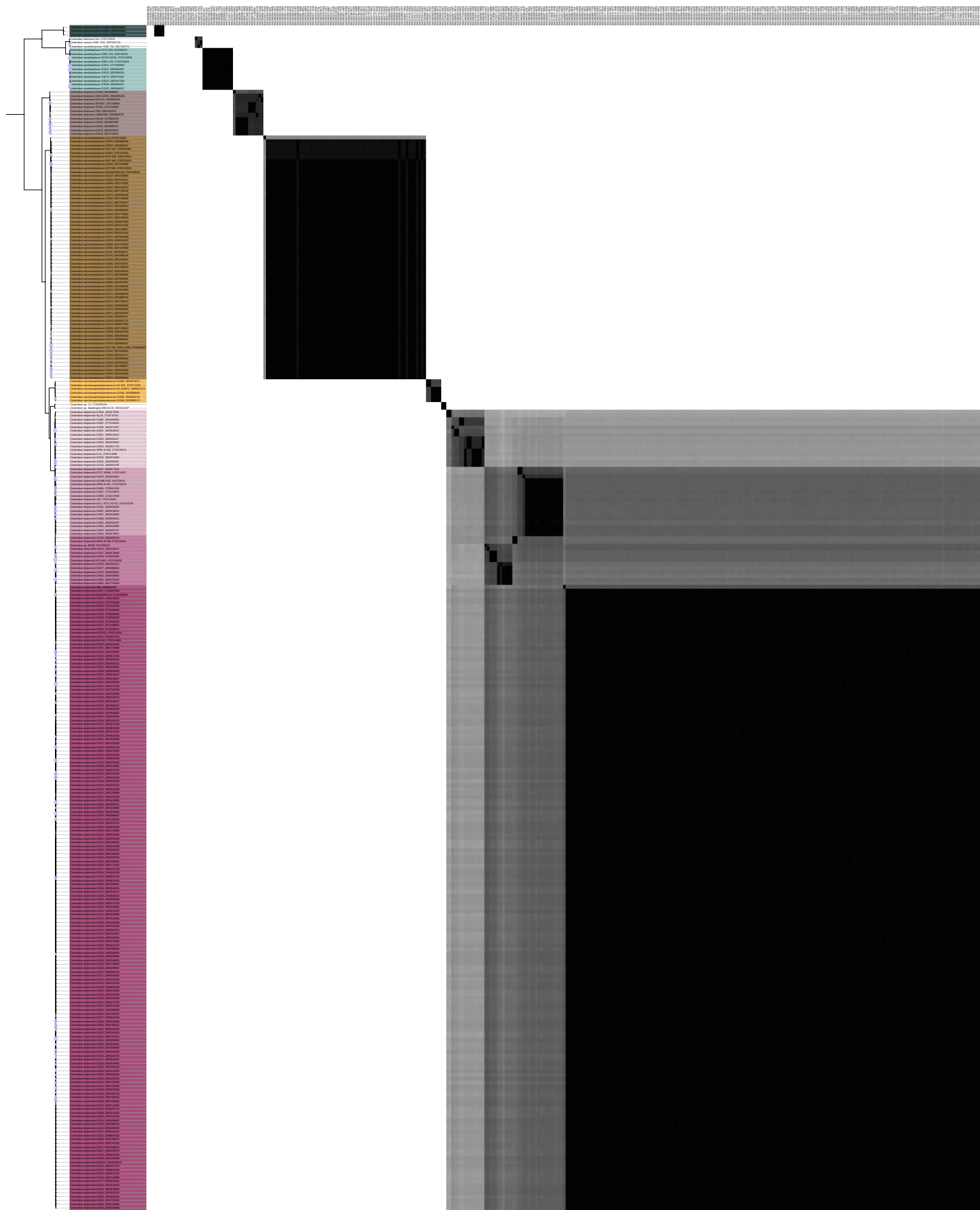

Supplement: Supplementary file 1 [file microorganisms-11-02253-s001.zip › microorganisms-2519127-supplementary/Supplamentary-Files/DJextended_ANI.pdf]
